# Data-Driven Motion Correction Algorithm: Validation in [^13^N]NH_3_ Dynamic PET/CT Scans

**DOI:** 10.3390/jcm15030984

**Published:** 2026-01-26

**Authors:** Oscar Isaac Mendoza-Ibañez, Riemer H. J. A. Slart, Charles Hayden, Tonantzin Samara Martínez-Lucio, Friso M. van der Zant, Remco J. J. Knol, Sergiy V. Lazarenko

**Affiliations:** 1 Department of Nuclear Medicine and Molecular Imaging, University Medical Center Groningen, University of Groningen, 9713 GZ Groningen, The Netherlands; 2 Department of Biomedical Photonic Imaging, University of Twente, 7522 NB Enschede, The Netherlands; 3 Molecular Imaging, Siemens Medical Solutions USA, Inc., Knoxville, TN 37932, USA; 4 Department of Nuclear Medicine, Northwest Clinics, 1815 JD Alkmaar, The Netherlands

**Keywords:** PET/CT, myocardial perfusion imaging, myocardial blood flow, coronary flow reserve, motion-correction, data-driven motion-correction, [^13^N]NH_3_

## Abstract

**Background**: Motion is a long-standing problem in cardiac PET/CT. An automated data-driven motion correction (DDMC) algorithm for within-reconstruction motion correction (MC) has been developed and validated in static images from [^13^N]NH_3_ and ^82^Rb PET/CT. This study aims to validate DDMC in dynamic [^13^N]NH_3_ PET/CT, and to explore the added value of DDMC in the evaluation of myocardial motion. **Methods**: Thirty-six PET/CT studies from normal patients and forty-three scans from patients with myocardial ischemia were processed using QPET software without MC (NMC), using manual in-software MC (ISMC), and DDMC. Differences in the mean values of rest-, stress-MBF, and CFR; and differences in effect size related to the use and type of MC method were explored. Moreover, motion vectors provided by DDMC were analyzed to evaluate differences in myocardial motion between scan phases and axes, and to elucidate changes in MBF quantification in relation to the motion extent. **Results**: In both subgroups, repeated measures ANOVA showed that the use of MC significantly increased regional and global stress-MBF and CFR values (*p* < 0.05), regardless of the MC method. Paired *t*-test analysis demonstrated a comparable ES between MC tools, despite minor differences in Cx, RCA and global rest-MBF values. High-intensity motion (>6 mm) proved to be present almost exclusively in the *Z* (cranio-caudal) direction. In the same axis, motion was significantly higher during stress than rest, regardless of patients’ subgroup. Finally, the Jonckheere trend test showed a significant trend caused by motion in s-MBF values, in which lower stress-MBF values were observed in response to motion extent increments. **Conclusions**: DDMC is feasible to perform in [^13^N]NH_3_ dynamic acquisitions and provides similar MBF/CFR values than manual ISMC. The use of DDMC reduces post-processing times and observer variability, and allows a more extensive evaluation of motion. MC is highly recommended when using QPET, as motion in the *Z*-axis during stress scans negatively impacts stress-MBF quantification.

## 1. Introduction

Quantitative evaluation of myocardial perfusion by dynamic PET/CT scans has an added value over conventional visual assessment from static PET/CT acquisitions [1,2]. Both values of myocardial blood flow (MBF) acquired during stress (s-MBF) and coronary flow reserve (CFR) [ratio of s-MBF/rest MBF (r-MBF)] have shown to act as prognostic markers in patients with proven or suspected coronary artery disease (CAD) [3,4,5,6,7].

Despite its advantages, MBF quantification still faces multiple pitfalls, including motion [8]. Motion impairs MPI PET/CT scans by interfering with the accurate tracking of radiotracer concentration over time, consequently diminishing the accuracy of the image-derived input function (IDIF) and the myocardial time–activity curves (TACs) that are used for MBF quantification via kinetic modeling [9]. Ultimately, motion negatively impacts the final reliability of MBF/CFR values [10,11]. Previous publications have demonstrated that motion in dynamic PET/CT scans is highly prevalent (30–68%), more prominent in the stress phase, affects the RCA territory more, and can be influenced by other factors, such as the stressor agent [12,13,14,15]. It has also been proposed that motion due to breathing is the most relevant source, as it is related to the appearance of “myocardial creep” (i.e., non-rigid displacement of the heart) [16]. Finally, it has been estimated that motion artifacts can introduce estimation errors of up to five times in the MBF/CFR values [17].

To overcome the presence of motion artifacts in cardiac scans, a wide range of motion correction (MC) tools have been proposed. Nevertheless, most of them deal only with the motion present in static images, and only a few can perform MC to dynamic acquisitions [18,19]. In a more recent effort, multiple vendors have incorporated built-in post-processing MC tools for the dynamic PET series [20,21,22]. Nevertheless, these approaches have an important limitation, in that they correct only for inter-frame motion while neglecting intra-frame motion. Another pitfall is that some of these MC tools must be implemented in a manual way, leading to time-consuming MC and arising concerns about inter- and intra-observer variability [23]. In recent years, a prototype data-driven motion correction (DDMC) tool which operates on raw PET data has emerged as a solution to perform within-reconstruction MC. This enables fast, robust, and reproducible MC; and benefits all outcomes’ series of cardiac PET/CT scans (i.e., static, gated, and dynamic). Furthermore, the motion vectors measured by DDMC can be analyzed, enabling a more extensive evaluation of the presence and extent of motion in scans. This algorithm has been previously validated for its use in static images from [^13^N]NH_3_ and ^82^Rb PET/CT examinations [24]. This study aims to validate the use of DDMC in dynamic acquisitions, using [^13^N]NH_3_ PET/CT scans; and to evaluate the added value of the high temporal resolution motion tracking performed by DDMC.

## 2. Materials and Methods

### 2.1. Study Population

Thirty-six [^13^N]NH_3_ PET/CT examinations from patients negative for myocardial ischemia or infarction, and forty-three patients positive for myocardial ischemia were retrospectively included from the clinical cohort of patients that underwent a [^13^N]NH_3_ PET/CT examination due to suspected IHD in the Northwest Clinics (Alkmaar, The Netherlands) between July 2020 and February 2023. Patients gave written informed consent to use their anonymized data. Besides the standard imaging protocol and clinical management, no additional measurements or actions affecting the patient were performed. The study was approved by the institutional research board; approval of the local ethical committee was not necessary since the study did not fall within the scope of the Dutch Medical Research Involving Human Subjects Act (Section 1.b, wet medisch-wetenschappelijk onderzoek [WMO], 26 February 1998).

### 2.2. Inclusion Criteria

Patients were classified into two groups according to the presence of myocardial ischemia. For selection of normal patients, the following inclusion criteria were applied: (1) no history of CAD; (2) PET/CT results in the PET/CT examination interpreted as normal by an experienced nuclear medicine physician, i.e., no significant perfusion defects by visual inspection of static images [i.e., normal summed stress score (SSS) and summed difference score (SDS)], LV-ejection fraction (LVEF) values >55% in the gated series, and global CFR interpreted as normal in the dynamic series; (3) diagnosis of CAD ruled out by medical consensus between nuclear medicine and cardiology departments, considering clinical and imaging evaluation; and (4) follow-up from the study date to the inclusion time without the presence of any major adverse cardiac event (MACE). In the case of patients positive for myocardial ischemia, the following inclusion criteria were used: (1) PET/CT results interpreted as suggestive of myocardial ischemia by an experienced nuclear medicine physician, i.e., significant perfusion defects by visual inspection of static images combined with abnormal values of SSS (≥4) and SDS (≥2), abnormal changes in LVEF between stress rest (<5%) in the gated series, and impaired regional s-MBF (<1.85 mL/g/min) and/or global CFR (<2) in the dynamic series; and (2) diagnosis of myocardial ischemia by consensus between the departments of nuclear medicine and cardiology after considering clinical and imaging evaluation.

### 2.3. Image Acquisition

**Figure 1:**
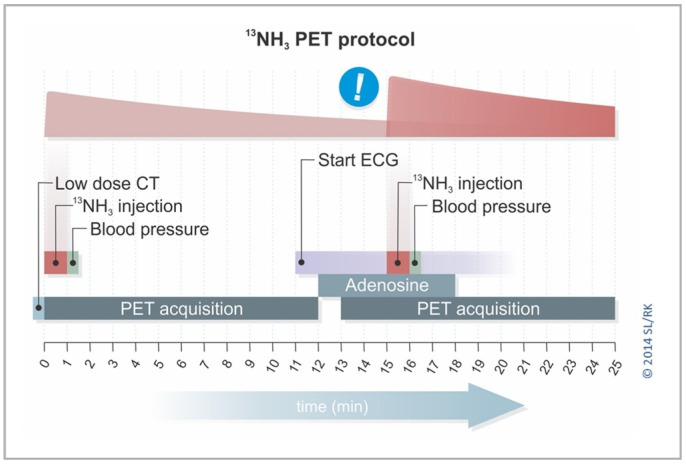
[^13^N]NH_3_ PET/CT time-efficient scanning protocol with two 12 min PET acquisitions (one during rest and one during pharmacological stress). Note how the scanning time before the second injection of [^13^N]NH_3_ is used for performing residual activity correction (as signaled by the exclamation sign with blue background).

All images were acquired using a Biograph Vision 600 PET/CT system (Siemens Healthineers, Knoxville, TN, USA). The complete protocol for image acquisition was reported in a previous publication from our group [25]. As shown in Figure 1, a 25 min time-efficient protocol, that implements a residual activity correction algorithm, is used. The protocol consists of a 12 min list-mode rest PET imaging acquisition using 300 MBq of [^13^N]NH_3_ in continuous infusion; and a 12 min stress imaging acquisition with 400 MBq of [^13^N]NH_3_-administered activity. Hyperemia was induced pharmacologically with either intravenous adenosine infusion (0.14 mg/kg/min for 6 min) or 400 µg regadenoson (bolus in 10 s, followed by 10 mL saline).

### 2.4. Image Reconstruction and Processing

Static, dynamic, and 16-bin ECG-gated images were generated from the list-mode data. The reconstruction of static, gated, and dynamic images was performed using PSF + TOF reconstruction, 4 iterations and 5 subsets, zoom 2, 220 × 220 matrix, and an isotropic Gaussian 3D filter of 4 mm. All reconstructed images were corrected for CT-based attenuation, scatter, decay, and random coincidences. Final images were verified for the presence of misalignment between the PET and CT data, and corrected manually before final reconstruction if necessary.

Dynamic rest images were reconstructed using the first 10 min of the rest acquisition data and using 25 frames (1 × 10, 12 × 5, 2 × 10, 7 × 30, 2 × 60, and 1 × 180 s), whereas dynamic stress images were reconstructed using the 10.5 min of data from stress acquisition after a delay of 90 s, using 26 frames (1 × 30, 1 × 10, 12 × 5, 2 × 10, 7 × 30, 2 × 60, and 1 × 180 s). Rest and stress dynamic data from every scan were reconstructed twice, first with the conventional reconstruction parameters described previously, and a second one where the list-mode data were first processed with the use of DDMC (version:2023a).

Image processing was performed with QPET software (Cedars-Sinai Medical Center, Los Angeles, CA, USA [version: 2018.0.0.232]). Images acquired from conventional reconstruction were processed with and without the use of the manual ISMC available in QPET, whereas the images retrieved from the DDMC reconstruction were processed only once, without the use of ISMC.

### 2.5. Manual Motion Correction

**Figure 2:**
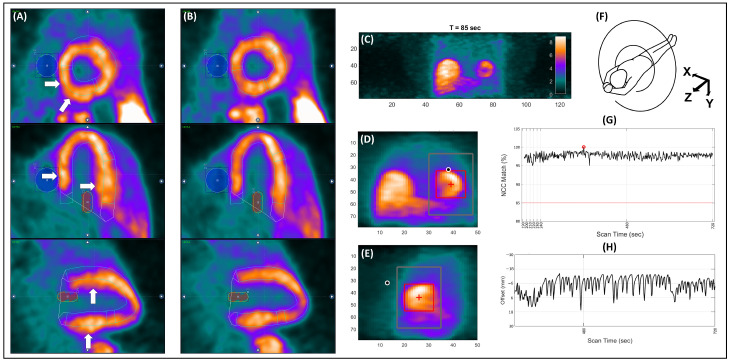
Methodology for motion correction of dynamic [^13^N]NH_3_ PET/CT scans using manual in-software motion correction (**A**,**B**) and data-driven motion correction (**C**–**H**). With the ISMC tool, the aim is to manually relocate the myocardial activity outside the myocardial contours [white solid arrows in (**A**)] to the inside of the myocardial borders (**B**). In the case of DDMC, the algorithm automatically constructs a 4D histo-image (**C**) for each second of acquisition time and further localizes a reference heart silhouette [red box in (**D**,**E**)]. Afterwards, according to the scanner alignment (**F**) and using a normalized correlation coefficients technique (**G**), DDMC draws a heart motion vector per second (**H**) for each axis (*X*, *Y* and *Z*).

QPET software incorporates manual frame-by-frame MC (ISMC), in which the operator can re-align short-axis, horizontal long-axis, and vertical long-axis images [20,22]. This ISMC tool must be independently applied for the rest and stress phases. For the realization of this project, QPET ISMC was applied in all patient scans, both in rest and stress acquisitions, and in all frames. In the blood pool frames (i.e., frames where radioactivity is located in the blood pool space but has not reached the myocardial tissue), the correction aimed to align the radiotracer activity to the endocardial borders of the LV cavity and to uniform the spillover activity. For the tissue-phase frames (i.e., frames where radiotracer activity is present in the myocardial tissue and no longer in the blood pool), the correction aimed to align the radiotracer activity within the LV myocardial contours. An example of how ISMC was performed for a later-phase frame in QPET is shown in Figure 2A,B.

### 2.6. Data-Driven Motion Correction

Descriptions of the DDMC software prototype’s operation have been previously published [24,25]. Figure 2C–H depict the functioning of DDMC. Briefly, this software bins the list-mode raw data file into 4D histo-images [3D space plus time], referred to as a “Direct Volume Histogram” (DVH) [Figure 2C], instead of binning it directly into a sinogram. After the construction of individual DVHs for each second of acquisition time (optimal frequency to create DVHs with the best trade-off between signal and noise [24]), the heart signature (REF) is located by searching for a relatively high-intensity area within a reference DVH from a period with high myocardial uptake and low extra-cardiac activity. In [^13^N]NH_3_ acquisitions, this reference DVH is most often located near the end of the acquisition. Afterwards, all the other DVH images are compared to the REF image for tracking the rigid heart movement in the 3D space. This comparison happens within a predefined search range in every DVH, referred to as SER [Figure 2D,E] and defines the *X*, *Y* and *Z* directions according to the scanner alignment (Figure 2F). A Normalized Cross Correlation (NCC) technique with an 85% threshold is used, in which the position with the highest NCC value indicates the heart’s location at any given time point (Figure 2G). Finally, MC is implemented during sinogram creation, producing a “motion-corrected” sinogram. In the current version, the DDMC prototype focuses on axial shifts and performs MC based on the motion vector from the *Z*-axis (Figure 2H). This setup has been implemented as cardiac motion occurs primarily in this direction, optimizes computational efficiency, and minimizes delays in obtaining corrected images.

### 2.7. Evaluation Metrics

Regional and global r-MBF, s-MBF, and CFR values were calculated in three different ways: (1) A conventional reconstruction process and image processing in QPET software without applying any MC tool [non-motion-corrected data (NMC data)]. (2) A conventional reconstruction process and image processing in QPET software with the use of the manual ISMC tool as already described (ISMC data). (3) Use of DDMC in the list-mode raw data, followed by a conventional reconstruction process and image processing in QPET software without the use of the QPET ISMC tool (DDMC data). Furthermore, the motion vectors in the *X*, *Y* and *Z* directions provided by the software were collected and used to compute a series of motion metrics. Each motion vector contained information regarding the displacement of the myocardial wall during each second of the dynamic acquisition with respect to a basal reference line of zero millimeters (mm) set by the software. These values were used to calculate the following measurements of motion per patient: mean myocardial displacement (MyoDis), and the number of seconds with displacement >3 mm, between 3 and 6 mm, between 6 and 9 mm, between 9 and 12 mm, and >12 mm. A three mm binning was decided on the basis that the approximate spatial resolution of the PET/CT scanner used in this project is of 3.2 mm. Finally, the MyoDis in the *Z*-axis was divided into tertiles, and patients were assigned into one of the following groups: ‘low motion’ (MyoDis ≤ first tertile), ‘mid motion’ (first tertile > MyoDis ≤ second tertile), or ‘high motion’ (MyoDis > second tertile). The Z direction was selected for patient categorization as, according to the literature, this is the direction with the highest motion; and since DDMC performs MC only in this direction.

### 2.8. Statistical Analysis

To evaluate if MC by DDMC was comparable to ISMC, two different approaches were used. In the first approach, differences related to the use and type of MC tool were evaluated by comparing regional and global mean values of r-MBF, s-MBF, and CFR acquired with NMC, ISMC, and DDMC using a repeated measures analysis of variance (RM-ANOVA) test. A pairwise *t*-test with Bonferroni correction for multiple comparisons was used for post hoc analysis in the case of a statistically significant RM-ANOVA. The second approach consisted of testing differences in effect size (ES) between MC tools. For this purpose, the ES for each MC tool was calculated by subtracting individual MBF/CFR values after the use of MC to the corresponding NMC MBF/CFR values. Differences in ES were compared using a paired *t*-test.

Values of MyoDis were demonstrated to follow a non-normal distribution. Hence, to test for differences in MyoDis related to the scan phase, a Mann–Whitney U test was used. For evaluating differences between axes, a Kruskal–Wallis test was used. A Dunn’s test with Bonferroni correction for multiple comparisons was used for post hoc analysis in the case of a statistically significant *p*-value in the Kruskal–Wallis test.

Differences in performance between DDMC and ISMC and differences in MyoDis were individually tested in the normal group and the myocardial ischemia group.

Finally, the Jonckheere–Terpstra test (also known as the Jonckheere trend test) was used to evaluate the effect of different amounts of motion in s-MBF values. This test was selected over a Kruskal–Wallis test, as an a priori hypothesis had been established that higher levels of motion would decrease final stress-MBF values. This analysis regarding the effect of motion was carried out using only the normal group when testing global s-MBF values, as the presence of obstructive lesion(s) in one or more coronary arteries was expected to interfere with the estimation of the effect of motion (i.e., a diminished global s-MBF in a patient with proven obstruction in the coronary arteries is not related to spill-in, spill-over or partial volume effects caused by the presence of cardiac motion). In the same way, when performing the analysis in the regional values of s-MBF, a composite population was used, consisting of all the vessels from the normal group and the vessels with no obstruction according to the invasive coronary angiography (ICA) report available for 16 of the 45 patients in the ischemic group. All other vessels with obstruction >30% or with no ICA information were not included in this analysis.

Before statistical analysis, the assumptions in the data for the realization of the statistical tests were checked. More specifically, for using a RM-ANOVA test, the data were evaluated for meeting the assumptions of normality (using quantile–quantile plots and the Shapiro–Wilk test) and sphericity (using Mauchly’s test). In the case of a paired *t*-test, data were evaluated for the assumption of normality before analysis. In the case of the Jonckheere–Terpstra test, it was ensured that the independence of observations and the a priori establishment of the order of the groups and direction of the alternative hypothesis. Statistical analyses were carried out using python 3.12.15 [26], statsmodels (v0.14.2) [27], and the pingouin (v0.5.5) [28] packages; and SPSS v29.0.1.0 (Armonk, NY, USA: IBM Corp.).

## 3. Results

### 3.1. Characteristics of Patients

Table 1 presents the characteristics of all patients included in the final analysis. It can be observed that the overall cohort consisted of elderly patients (>65 years old), most of them stressed with adenosine (69.6%), and the rest with regadenoson. Some differences, related to the underlying diagnosis, can be observed between the group of normal patients and patients with myocardial ischemia. More specifically, there was a different proportion of male and female individuals, with the female gender more prevalent in normals and the male gender in ischemic. Also, mean values of global s-MBF and CFR were lower in the ischemic group.

### 3.2. Effect of MC Tools on MBF Quantification

The effect of the use of MC in regional and global MBF values from normal and ischemic patients are presented in Table 2 and Table 3, respectively. The results from RM-ANOVA indicated that, regardless of the patient group and MC tool, the use of MC led to the obtention of significantly higher regional and global s-MBF and CFR values than NMC values. Additionally, the analysis shows that similar s-MBF and CFR values are obtained with the use of any of the MC methods, as no significant differences were found between values from ISMC and DDMC. In r-MBF, the effect of MC was heterogeneous and differed between patient group and MC tool. While the use of DDMC did not change significantly any regional or global r-MBF value in the normal or ischemic group, the use of ISMC led to the obtention of significantly higher r-MBF values in the circumflex (Cx) region in both groups. Finally, in the ischemic group, r-MBF values in the RCA region were shown to be significantly different if acquired with the use of ISMC or DDMC. Nevertheless, it must be mentioned that the mean difference in r-MBF values was marginal in both groups, with a maximum mean difference of 0.05 mL/g/min (RCA region of the normal group).

### 3.3. Effect-Size of MC Tools

**Figure 3:**
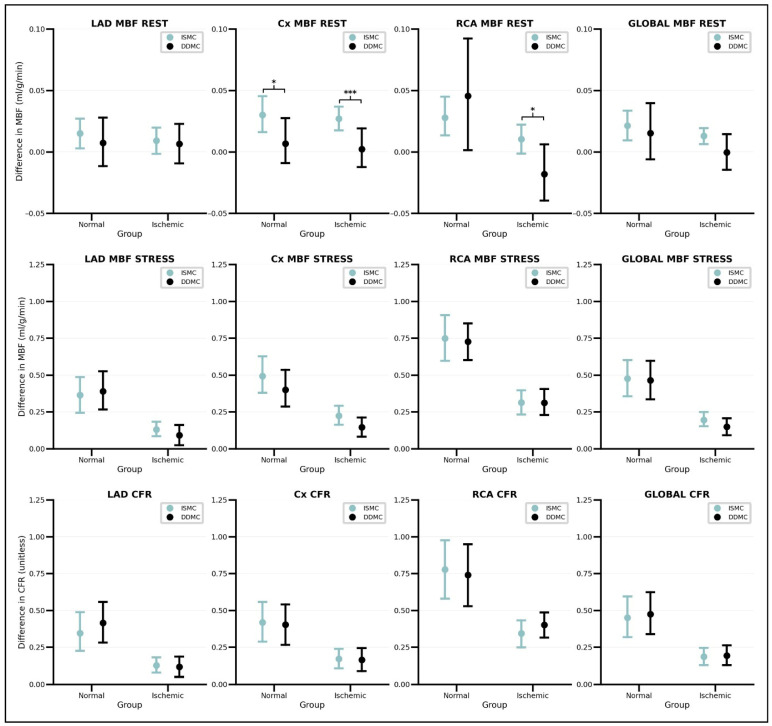
Effect sizes of the in-software motion correction tool and data-driven motion correction algorithm. Note how the effect size is considerably lower for regional and global r-MBF values when compared to s-MBF and CFR values. Also note how it is only during rest that significant differences in the ES are found when comparing between the methods (significant *p*-value signaled with asterisks [*p* < 0.05 = * and *p* < 0.001 = ***]).

Differences in ES between ISMC and DDMC, both for the normal and ischemic groups, are presented in Figure 3. It can be observed that ISMC and DDMC showed significantly different ESs only in r-MBF values. This was the case for r-MBF values in the Cx and RCA territories of ischemic patients and the Cx territory in the normal group. Nevertheless, it must be highlighted that the overall ES of the MC tools in r-MBF was minimal, considering a mean ES of 0.05 mL/g/min that represents a change of only 5% when compared to the mean r-MBF with NMC in our cohort of approximately 1.00 mL/g/min. The ES of the use of MC on s-MBF and CFR values proved to be similar between ISMC and DDMC, irrespective of the region being studied or the patient group. Contrary to what was observed in r-MBF, substantially large ESs were noticed, particularly in the group of normal patients and the RCA and global regions, where mean ESs up to 0.78 mL/g/min in the RCA s-MBF variable were obtained. These ESs represent a mean change of up to 34.8% when compared to a mean RCA s-MBF value of 2.24 mL/g/min obtained without the use of MC.

### 3.4. DDMC Motion Tracking per Second

**Figure 4:**
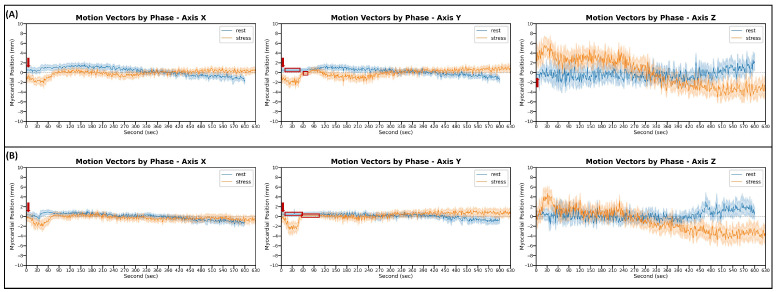
Average motion vectors as measured by DDMC in the 3D space of scans from normal patients (**A**) and patients with myocardial ischemia (**B**). It can be observed that heart displacement is markedly higher in the cranio-caudal direction (*Z*-axis), regardless of the phase, in both (**A**) and (**B**). In the *Z*-axis, it can also be observed how the myocardial wall is always further from the reference zero line (gray dotted line) during stress (orange solid line) than in rest (blue solid line). Red arrows and red boxes indicate the periods where DDMC was unbale to track the myocardial wall position at the beginning of rest acquisitions and during the blood-pool phase of *Y*-axis motion vectors, respectively.

Figure 4 illustrates the averaged motion vector (with its corresponding 95% confidence interval) for every patient group, phase, and axis, as measured by DDMC. It is visually evident that myocardial motion appears to be higher in the *Z*-axis, regardless of the phase and patient group. The red arrows in Figure 4 show a lack of motion tracking for a brief period at the beginning of rest scans. The red boxes show a lack of motion tracking in the *Y* direction in the early phase of the motion vectors from rest and stress scans. A lack of tracking at the start of rest scans is explained by the rest acquisition starting simultaneously with the [^13^N]NH_3_ injection. As DDMC tracks the myocardial wall based on the histo-images constructed with the emission data (i.e., radioactivity) present in the body, the algorithm is unable to locate the myocardium during the seconds where the tracer has not reached the heart. During stress acquisitions, this issue is avoided, as there is still residual radioactivity from the rest scan due to the nature of the time-efficient protocol. The lack of tracking in the early phase of *Y*-axis motion vectors is an intentional configuration of the software. DDMC uses two image planes for motion tracking: a coronal view (Figure 2D) from which motion in the *X* and *Z* directions is estimated; and a sagittal view (Figure 2E), from which motion in the *Y* direction is tracked. Motion tracking in the sagittal view is omitted during the bolus phase (i.e., blood pool phase) to maintain computational efficiency and not introduce significant delays to the total reconstruction time of scans; as during this phase, other solutions must be applied (e.g., refreshing the reference DVH, use of a high-pass filter).

### 3.5. Amount of Motion as Measured by DDMC

**Figure 5:**
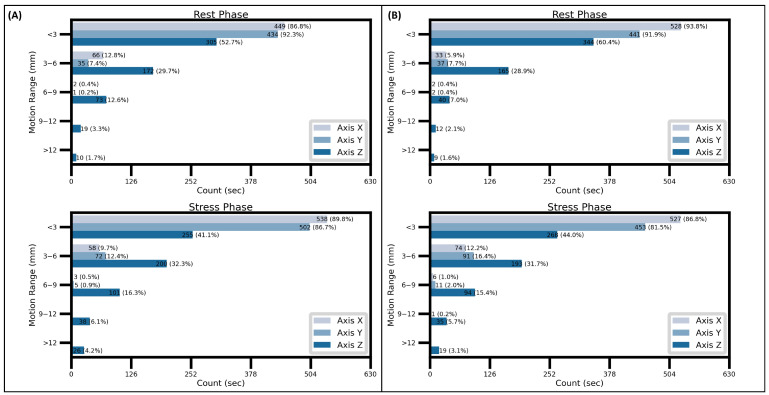
Average amount of motion of different ranges (i.e., intensities) in the 3D space. Note that both in the normal (**A**) and ischemic (**B**) groups, motion > 6 mm in intensity is negligible in the *X* and *Y* axes, whereas in the *Z*-axis there is a substantial amount of motion > 6 mm, particularly during the stress phase.

As shown in Figure 5, individual values of myocardial displacement per second were categorized into five different motion intensity categories. It can be observed that, on average, and regardless of the patient group and phase, motion of 6 mm or higher is practically negligible in the *X-* and *Y*-axis (less than 2% of the average total time with motion tracking). In contrast, in the *Z*-axis motion > 6 mm was present, on average, in up to 26.6% of the motion-tracked time. It must also be noted that there was a substantial difference in the average time with motion > 6 mm between stress and rest scans. In normal patients (Figure 5A), motion > 6 mm was present 26.6% of the time, versus only 17.6% during rest, representing a difference of 63 sec of motion-tracked time. In the patients with ischemia (Figure 5B), a similar behavior was observed, with a difference of 13.5% (87 sec of motion-tracked time) in motion > 6 mm between stress and rest phases [24.2% (stress), 10.7% (rest)].

**Figure 6:**
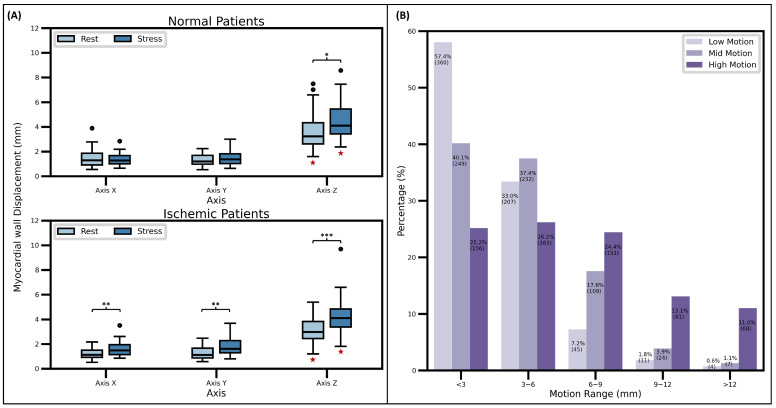
Myocardial wall displacement (MyoDis) metrics. Note how, according to (**A**), the MyoDis is significantly higher in the *Z*-axis regardless of the patient group and phase (red stars); and MyoDis is significantly higher during stress for most of the axis (signaled by asterisks reflecting *p*-values < 0.05 = *, <0.01 = ** and <0.001 = ***). When using the percentiles of MyoDis, the *Z*-axis for classification of patients into groups of low, mid and high motion (**B**), it can be noticed the substantial difference in the amount of motion < 6 mm, that goes from a 90% prevalence in the “low motion” group to only 51% prevalence in the “high motion” group.

Also, as described earlier in the text, individual values of myocardial displacement per second were averaged to estimate the MyoDis per patient. Figure 6A displays the mean MyoDis values for every phase and axis in both patient groups. It can be observed that the MyoDis in the *Z*-axis was significantly higher than for the other two axes, irrespective of the scan phase (signaled by the red star under the specific boxplots). In the same line, MyoDis during stress is demonstrated to be significantly higher, regardless of the patient group, in the same *Z*-axis (signaled by asterisks in the respective axis). Some differences were observed between normal and ischemic patients. More specifically, in the ischemic group MyoDis in stress proved to be significantly higher than in rest, something not observed in the normal group. Despite these differences, the overall MyoDis in the *X* and *Y* axes was small, with a maximum median MyoDis value of 1.68 mm.

The amount of MyoDis in the *Z*-axis was used to classify patients into “low motion”, “mid motion” and “high motion” categories. As shown in Figure 6B, MyoDis is demonstrated to be closely related to the motion intensity during the motion-tracked time. For instance, if a cut-off value of 6 mm is used, it can be observed that the prevalence of motion <6 mm gradually decreases from 90.4% in the “low motion” group, to 77.5% in the “mid motion” group, and to only 51.4% in the “high Motion” group. Conversely, motion >6 mm gradually increases from 9.6% to 22.5% and to 48.6%, in the “low motion”, “mid motion” and “high motion” groups, respectively.

### 3.6. Effect of the Extent of Motion in MBF Quantification

**Figure 7:**
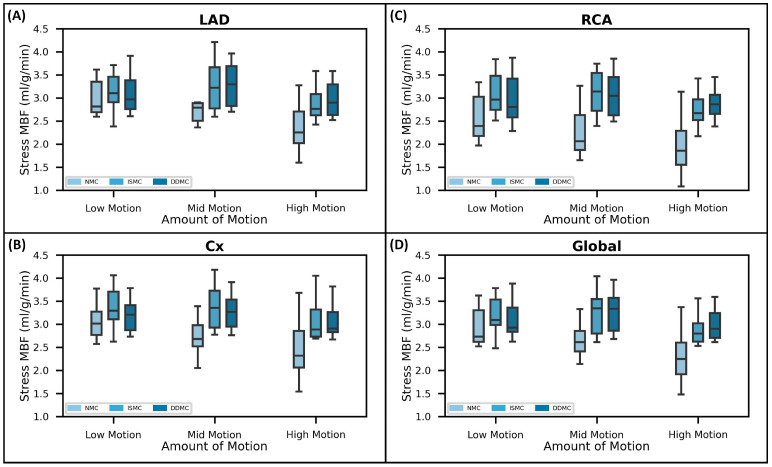
Effect of motion extent in the *Z*-axis during stress in stress-MBF values. Note how both at the regional (**A**–**C**) and global (**D**) level, a higher motion extent is related to lower stress MBF values. Note also how this significant trend disappears once that motion correction is performed, either with ISMC or DDMC.

When relating the motion extent in the *Z*-axis during stress to s-MBF values, a statistically significant negative trend in MBF values was found in relation to increments in the motion extent. This is illustrated in Figure 7, where it can be observed how regional and global s-MBF values with NMC gradually diminished as they passed from the “low motion” to the “high motion” group. Once MC is applied, this trend becomes not significant, as observed in Figure 7 when observing regional and global s-MBF values obtained with the use of either ISMC or DDMC.

## 4. Discussion

This work presents a comprehensive evaluation of a DDMC algorithm for within-reconstruction MC in dynamic [^13^N]NH_3_ cardiac PET/CT acquisitions with the objective of validating its use in this series. Our main results prove that dynamic MC using DDMC is comparable to ISMC, as the use of either MC method produced a similar effect in MBF quantification by leading the obtention of statistically significant higher s-MBF and CFR values both at the regional and global level. Moreover, the values of s-MBF and CFR proved not to be different between ISMC and DDMC, and finally, all findings remained constant among patients with and without myocardial ischemia. Although some significant differences were observed in mean r-MBF values when acquired with ISMC and DDMC, further analysis of the effect size demonstrated that the overall effect of MC tools in the rest phase is clinically irrelevant (<0.05 mL/g/min of ES for both MC tools). Similar results can be found in the literature. For instance, Lee et al. (2020) [21] reported several significant differences in r-MBF combined with overall small ESs (<0.02 mL/g/min) in a research project aiming to evaluate the performance of a new dynamic MC approach (i.e., automated frame-by-frame MC against manual frame-by-frame MC). However, contrary to our findings, in most of the regional and global s-MBF and CFR values, the use of MC, either automated or manual, led to the obtention of lower MBF/CFR values. Additionally, their results showed an ES in s-MBF and CFR values considerably lower than the ESs reported in our work. A possible explanation of this discrepancy is the dependency of MBF quantification on age, gender, vascular territory, and software package used for image processing, as described by Sunderland et al. [29]. Consequently, it can be expected that the impact of motion and the effect of MC in MBF quantification are also dependent on these factors. The use of a different software package for image processing by Lee and colleagues could have a major role in explaining these differences in the reported findings. Additionally, the lack of a true reference standard when it comes to motion measuring and MBF/CFR quantification, as highlighted by Votaw et al., adds more complexity when trying to elaborate robust and reproducible MC methods, and when trying to compare between them. Finally, and on the same topic of software dependency, our results have demonstrated how critical it is to use MC in the context of dynamic [^13^N]NH_3_ PET/CT processing with the use of QPET, particularly for s-MBF and CFR values, a fact that must be considered when using this software package. Considering this finding, further research specifically aimed at elucidating the sensitivity to motion of software programs commonly used for dynamic [^13^N]NH_3_ image processing from other vendors must be conducted.

In this project, manual ISMC was used as a reference standard for comparison of the expected performance for a new MC method. However, it must be noted that both tools present important setup differences, and, in this way, an exact equal performance should not be expected. First, as mentioned earlier in the text, manual ISMC in QPET is performed in every temporal frame by shifting short-axis, horizontal long-axis, and vertical long-axis images. Hence, ISMC is limited to correct only frame-by-frame motion. ISMC could be considered a MC technique in the 3D space (*X*, *Y* and *Z* directions), assuming the user performed a visual inspection and correction, if necessary, in all axis views of every frame of rest and stress acquisitions. However, it is difficult for this assumption to be fulfilled. Christensen and colleagues [12] have shown that when motion is assessed visually, the extent of motion is easily under- or overestimated. Furthermore, they showed that motion in the *Z*-axis was more easily identifiable and corrections in that direction outweighed those in the other axes. On the other hand, DDMC tracks the rigid heart wall motion using the list-mode raw data during the total time of rest and stress acquisitions, in the 3D space and at one-second resolution. However, only the motion in the *Z*-axis is incorporated into the process of sinogram binning to deliver a motion-corrected sinogram. In this way, DDMC could be considered a 3D motion tracking tool but a 1D motion correction tool. Furthermore, DDMC represents a “within-reconstruction” MC tool, that detects motion at a high temporal resolution and does not neglect intra-frame motion. Although both MC tools evaluated for the purposes of this project were demonstrated to be comparable for MC of dynamic [^13^N]NH_3_ acquisitions, the use of DDMC ultimately implies some advantages when compared to either conventional manual or automated frame-by-frame MC tools. First, as stated by Rubeaux et al. [30], it represents a “desirable” MC method, as within-reconstruction MC tools have been shown to have less noise and bias in images than post-reconstruction approaches. Secondly, although this paper has only evaluated the effect of DDMC in dynamic acquisitions, DDMC performs simultaneous within-reconstruction MC to all acquisition series of cardiac PET/CT scans (i.e., static, gated, and dynamic series). This is something generally uncommon for MC tools, as most MC tools are specifically developed to work exclusively with one type of series data. Previous publications have demonstrated that the use of DDMC is able to improve the image quality of static images, and “rescue” cardiac PET/CT scans that, without proper MC, would have been of no clinical utility [31]. Also, the pitfall of inter- and intra-observer variability must also be considered when using manual MC tools, a problem that is avoided with DDMC. And finally, the added time represented by performing manual MC has to be considered. In our experience, correcting a whole PET/CT examination can take between 5 and 12 min, depending on the experience and familiarity of the user with the software package, but also on the motion burden present in the scan. Despite all the advantages of DDMC, probably the most desirable approach for dealing with motion in dynamic PET/CT scans would be a combination of MC tools, where a standardized within-reconstruction MC method (such as the DDMC protype) is applied routinely to all scans, but regardless, all scans are further inspected post-reconstruction and proper manual MC tools are available for performing further refinements if needed. The latest efforts have implemented the use of deep-learning algorithms to develop automatic MC tools [32,33]; nevertheless, this novel approach still presents similar limitations when compared to non-AI automatic MC tools, mainly regarding the issue of dealing only with frame-by-frame motion and not correcting for intra-frame motion.

Finally, our analysis has also demonstrated the added value of the motion tracking per second with DDMC when analyzing the motion burden in dynamic [^13^N]NH_3_ acquisitions. By using the motion vectors in the 3D space provided by DDMC, we have been able to describe the rigid myocardial displacement in a highly detailed manner. Also, using this approach, we have avoided the pitfall of human observations (subjectiveness and observer variability) for the detection and estimation of motion. It is difficult to compare our findings to what previous publications have reported, as most of the previous publications have relied on visual inspection and have evaluated only frame-by-frame motion. We have reported that motion in the *X* and *Y* directions is negligible, as it remains lower than 3 mm for ≈80% of the dynamic acquisition time, regardless of the phase or patient group (i.e., normal or with ischemia). Also, we have demonstrated that motion is higher during stress, particularly in the *Z*-axis. In the *X* and *Y* directions, higher motion during stress was observed only for the group with myocardial ischemia. Our findings in the *Z*-axis are in line with what was previously reported by Vleeming et al. [34], when examining the inter-frame myocardial 3D motion adenosine and regadenoson scans. In their study, they found the *Z*-axis as the direction with the highest displacement, regardless of the stressor agent [mean maximum displacements during stress of 2.5 mm (*X*-axis), 4.8 mm (*Y*-axis), and 9.9 mm (*Z*-axis) in adenosine scans; and of 2.9 mm (*X*-axis), 3.1 mm (*Y*-axis), and 7.1 mm (*Z*-axis) in regadenoson scans]. Other publications, such as the research project conducted by Hunter et al. [17], have reported the highest prevalence of motion in the vertical direction, even when evaluated using a visual approach. Some discrepancies are present in the literature regarding the motion extent present in rest and stress scan phases. For instance, Hunter et al. [17] reported no significant differences in motion between rest and stress phases. However, both Lee et al. [35] and Vleeming and colleagues [34] have described more motion during stress. Previous publications have also suggested different extents of motion in relation to different periods of scans. For instance, Lee et al. [35] have reported that motion is minimal after two minutes post-injection, while Hunter and colleagues [17] reported motion constant in frequency up to minute eight of acquisition. This discrepancy has been suggested to be in relation to the reference segmentation, where, if the reference myocardial contours are derived from late-phase images, it is easy to interpret that motion is present in the early phase [36]. Finally, it has been described that motion is also related to the stressor agent, with higher motion described when using adenosine [34,36]. Nevertheless, we did not test differences in motion between adenosine and regadenoson scans due to the low number of scans performed with regadenoson (30% in the overall cohort).

## 5. Conclusions

DDMC is feasible to perform in [^13^N]NH_3_ dynamic acquisitions and shows a comparable performance to manual ISMC. DDMC allows for a reduction in post-processing times and observer variability inherent to manual MC and has the potential to enable a more reproducible and extensive evaluation of motion. MC is highly recommended when using QPET for image processing, as significantly lower stress-MBF and CFR values are retrieved when not using MC. In this software package, the amount of motion in the *Z*-axis during stress is related to a negative trend in s-MBF values, in which, as motion increases, regional/global s-MBF values decrease.

## 6. Limitations

This study presents certain limitations that must be addressed. First, the sample size is relatively small when compared to similar studies on the topic that have included over 200 patients. Second, DDMC processing still presents a major limitation in motion tracking related to computational efficiency, as DDMC does not perform motion tracking in the *Y* direction during the blood pool phase of the acquisition and, consequently, these seconds are not included in the final analysis. Although these seconds represent a minor percentage in relation to the total acquisition time, it may be possible that there is an underestimation of the motion extent in the *Y* direction. Future refinements to DDMC must include the incorporation of the *Y*-axis within the motion tracking process for the entirety of scan acquisitions. Finally, motion appears to be highly related to the software package used for image processing. Hence, the effect size of the MC methods and the effect of motion in MBF values are expected to differ from what is reported in this publication with the use of different software packages.

## Figures and Tables

**Table 1:** Patients’ characteristics.

Characteristic	Normal Group n = 36	Ischemic Group n = 43
Sex—n (%)	10/36 male (28%)	31/43 male (70.5%)
Age—mean (SD)	66 years (±12)	69 years (±8)
BMI—mean (SD)	27.2 kg/m^2^ (±4.40)	28.0 kg/m^2^ (±8.49)
Stress-agent—n (%)	Adenosine: 29 (81%)	Adenosine: 26 (60%)
	Regadenoson: 7 (19%)	Regadenoson: 17 (40%)
Global MBF in rest—mean (±SD)	1.02 mL/g/min (±0.27)	0.94 mL/g/min (±0.23)
Global MBF in stress—mean (±SD)	2.65 mL/g/min (±0.52)	2.01 mL/g/min (±0.55)
Global CFR—mean (±SD)	2.72 mL/g/min (±0.70)	2.21 mL/g/min (±0.60)

**Table 2:** MBF values by MC approach in normal patients.

Variable	Mean Value NMC (±SD)	Mean Value ISMC (±SD)	Mean Value DDMC (±SD)	Differences in MBF/CFR Values Related to MC Tool	*p*-Value (RM-ANOVA)	NMC vs. ISMC	*p*-Value (Paired *t*-Test)	NMC vs. DDMC	*p*-Value (Paired *t*-Test)	ISMC vs. DDMC	*p*-Value (Paired *t*-Test)
LAD MBF Rest (mL/g/min)	1.01 (±0.27)	1.03 (±0.27)	1.02 (±0.29)	No	ns	-	-	-	-	-	-
Cx MBF Rest (mL/g/min)	1.06 (±0.26)	1.09 (±0.28)	1.06 (±0.29)	Yes	<0.01	Yes	<0.01	No	ns	No	ns
RCA MBF Rest (mL/g/min)	0.95 (±0.28)	0.98 (±0.28)	1.00 (±0.32)	No	ns	-	-	-	-	-	-
GLOBAL MBF Rest (mL/g/min)	1.02 (±0.27)	1.04 (±0.27)	1.03 (±0.29)	No	ns	-	-	-	-	-	-
LAD MBF Stress (mL/g/min)	2.73 (±0.51)	3.09 (±0.46)	3.12 (±0.42)	Yes	<0.001	Yes	<0.001	Yes	<0.001	No	ns
Cx MBF Stress (mL/g/min)	2.78 (±0.54)	3.27 (±0.47)	3.18 (±0.36)	Yes	<0.001	Yes	<0.001	Yes	<0.001	No	ns
RCA MBF Stress (mL/g/min)	2.24 (±0.57)	2.98 (±0.45)	2.96 (±0.42)	Yes	<0.001	Yes	<0.001	Yes	<0.001	No	ns
GLOBAL MBF Stress (mL/g/min)	2.65 (±0.52)	3.12 (±0.44)	3.11 (±0.38)	Yes	<0.001	Yes	<0.001	Yes	<0.001	No	ns
LAD CFR (unitless)	2.81 (±0.70)	3.16 (±0.70)	3.22 (±0.70)	Yes	<0.001	Yes	<0.001	Yes	<0.001	No	ns
Cx CFR (unitless)	2.74 (±0.69)	3.15 (±0.68)	3.14 (±0.64)	Yes	<0.001	Yes	<0.001	Yes	<0.001	No	ns
RCA CFR (unitless)	2.45 (±0.76)	3.23 (±0.79)	3.19 (±0.81)	Yes	<0.001	Yes	<0.001	Yes	<0.001	No	ns
GLOBAL CFR (unitless)	2.72 (±0.70)	3.17 (±0.70)	3.19 (±0.68)	Yes	<0.001	Yes	<0.001	Yes	<0.001	No	ns

SD = standard deviation, NMC = no motion correction, ISMC = in-software motion correction, DDMC = data-driven motion correction, MBF = myocardial blood flow, CFR = coronary flow reserve, ns = non-significant.

**Table 3:** MBF values by MC approach in ischemic patients.

Variable	Mean Value NMC (±SD)	Mean Value ISMC (±SD)	Mean Value DDMC (±SD)	Differences in MBF/CFR Values Related to MC Tool	*p*-Value (RM-ANOVA)	NMC vs. ISMC	*p*-Value (Paired *t*-Test)	NMC vs. DDMC	*p*-Value (Paired *t*-Test)	ISMC vs. DDMC	*p*-Value (Paired *t*-Test)
LAD MBF Rest (mL/g/min)	0.93 (±0.24)	0.93 (±0.23)	0.93 (±0.24)	No	ns	-	-	-	-	-	-
Cx MBF Rest (mL/g/min)	0.98 (±0.26)	1.01 (±0.26)	0.99 (±0.26)	Yes	0.001	Yes	<0.001	No	ns	Yes	<0.05
RCA MBF Rest (mL/g/min)	0.90 (±0.22)	0.91 (±0.23)	0.88 (±0.23)	Yes	<0.05	No	ns	No	ns	Yes	<0.05
GLOBAL MBF Rest (mL/g/min)	0.94 (±0.23)	0.95 (±0.23)	0.94 (±0.23)	No	ns	-	-	-	-	-	-
LAD MBF Stress (mL/g/min)	2.04 (±0.61)	2.17 (±0.66)	2.13 (±0.59)	Yes	<0.001	Yes	<0.001	Yes	<0.05	No	ns
Cx MBF Stress (mL/g/min)	2.21 (±0.62)	2.44 (±0.64)	2.36 (±0.65)	Yes	<0.001	Yes	<0.001	Yes	<0.001	No	ns
RCA MBF Stress (mL/g/min)	1.71 (±0.63)	2.02 (±0.74)	2.02 (±0.76)	Yes	<0.001	Yes	<0.001	Yes	<0.001	No	ns
GLOBAL MBF Stress (mL/g/min)	2.01 (±0.55)	2.21 (±0.59)	2.16 (±0.55)	Yes	<0.001	Yes	<0.001	Yes	<0.001	No	ns
LAD CFR (unitless)	2.28 (±0.69)	2.41 (±0.75)	2.40 (±0.78)	Yes	<0.001	Yes	<0.001	Yes	<0.01	No	ns
Cx CFR (unitless)	2.34 (±0.65)	2.51 (±0.69)	2.50 (±0.76)	Yes	<0.001	Yes	<0.001	Yes	<0.001	No	ns
RCA CFR (unitless)	1.93 (±0.68)	2.28 (±0.77)	2.34 (±0.82)	Yes	<0.001	Yes	<0.001	Yes	<0.001	No	ns
GLOBAL CFR (unitless)	2.21 (±0.60)	2.40 (±0.65)	2.41 (±0.70)	Yes	<0.001	Yes	<0.001	Yes	<0.001	No	ns

SD = standard deviation, NMC = no motion correction, ISMC = in-software motion correction, DDMC = data-driven motion correction, MBF = myocardial blood flow, CFR = coronary flow reserve, ns = non-significant.

## Data Availability

The data presented in this study are available on request from the corresponding author due to privacy and ethical restrictions.

## Abbreviations

The following abbreviations are used in this manuscript:

PET/CT	Positron emission tomography/computed tomography
MBF	Myocardial blood flow
CFR	Coronary flow reserve
MC	Motion correction
DDMC	Data-driven motion correction
[^13^N]NH_3_	^13^N-labeled ammonia
ISMC	In-software motion correction
